# The Role of the Kynurenine Pathway in the (Patho) physiology of Maternal Pregnancy and Fetal Outcomes: A Systematic Review

**DOI:** 10.1177/11786469221135545

**Published:** 2022-11-30

**Authors:** Sofie KM van Zundert, Michelle Broekhuizen, Ashley JP Smit, Lenie van Rossem, Mina Mirzaian, Sten P Willemsen, AH Jan Danser, Yolanda B De Rijke, Irwin KM Reiss, Daphne Merkus, Régine PM Steegers-Theunissen

**Affiliations:** 1 Department of Obstetrics and Gynecology, Erasmus MC University Medical Center, Rotterdam, The Netherlands; 2 Department of Clinical Chemistry, Erasmus MC University Medical Center, Rotterdam, The Netherlands; 3 Division of Neonatology, Department of Pediatrics, Erasmus MC University Medical Center, Rotterdam, The Netherlands; 4 Division of Pharmacology and Vascular Medicine, Department of Internal Medicine, Erasmus MC University Medical Center, Rotterdam, The Netherlands; 5 Division of Experimental Cardiology, Department of Cardiology, Erasmus MC University Medical Center, Rotterdam, The Netherlands; 6 Department of Biostatistics, Erasmus MC University Medical Center, Rotterdam, The Netherlands; 7 Walter Brendel Center of Experimental Medicine, University Clinic Munich, Ludwig Maximillian University Munich, Munich, Germany

**Keywords:** tryptophan, pregnancy, depression, gestational diabetes mellitus, preeclampsia, pregnancy-induced hypertension, fetal growth restriction, preterm birth, spontaneous abortion

## Abstract

**Introduction:**

Tryptophan is the precursor of kynurenine pathway (KP) metabolites which regulate immune tolerance, energy metabolism, and vascular tone. Since these processes are important during pregnancy, changes in KP metabolite concentrations may play a role in the pathophysiology of pregnancy complications. We hypothesize that KP metabolites can serve as novel biomarkers and preventive therapeutic targets. This review aimed to provide more insight into associations between KP metabolite concentrations in maternal and fetal blood, and in the placenta, and adverse maternal pregnancy and fetal outcomes.

**Methods:**

A systematic search was performed on 18 February 2022 comprising all KP metabolites, and keywords related to maternal pregnancy and fetal outcomes. English-written human studies measuring KP metabolite(s) in maternal or fetal blood or in the placenta in relation to pregnancy complications, were included. Methodological quality was assessed using the ErasmusAGE quality score (QS) (range: 0-10). A meta-analysis of the mean maternal tryptophan and kynurenine concentrations in uncomplicated pregnancies was conducted.

**Results:**

Of the 6262 unique records, 37 were included (median QS = 5). Tryptophan was investigated in most studies, followed by kynurenine, predominantly in maternal blood (n = 28/37), and in the second and third trimester of pregnancy (n = 29/37). Compared to uncomplicated pregnancies, decreased tryptophan in maternal blood was associated with an increased prevalence of depression, gestational diabetes mellitus, fetal growth restriction, spontaneous abortion, and preterm birth. Elevated tryptophan was only observed in women with pregnancy-induced hypertension compared to normotensive pregnant women. In women with preeclampsia, only kynurenic acid was altered; elevated in the first trimester of pregnancy, and positively associated with proteinuria in the third trimester of pregnancy.

**Conclusions:**

KP metabolite concentrations were altered in a variety of maternal pregnancy and fetal complications. This review implies that physiological pregnancy requires a tight balance of KP metabolites, and that disturbances in either direction are associated with adverse maternal pregnancy and fetal outcomes.

## Introduction

The essential amino acid tryptophan is required for protein synthesis, and is therefore important for growth and development of the placenta and fetus. Tryptophan is also the substrate for multiple metabolic pathways, including the serotonin pathway, tryptamine pathway and indole pathway.^1^ However, by far the greatest proportion of tryptophan (>95%) is metabolized via the kynurenine pathway (KP). KP metabolites have pro- and antioxidant effects and are involved in many physiological processes that play a key role in pregnancy, including the regulation of vascular tone in the mother and placenta, immune tolerance, and neuroprotection.^2,3^ Indeed, the KP, and equally important, the transport of its metabolites across the placenta, affect placental function and pregnancy outcome.^4,5^

**Figure 1.**
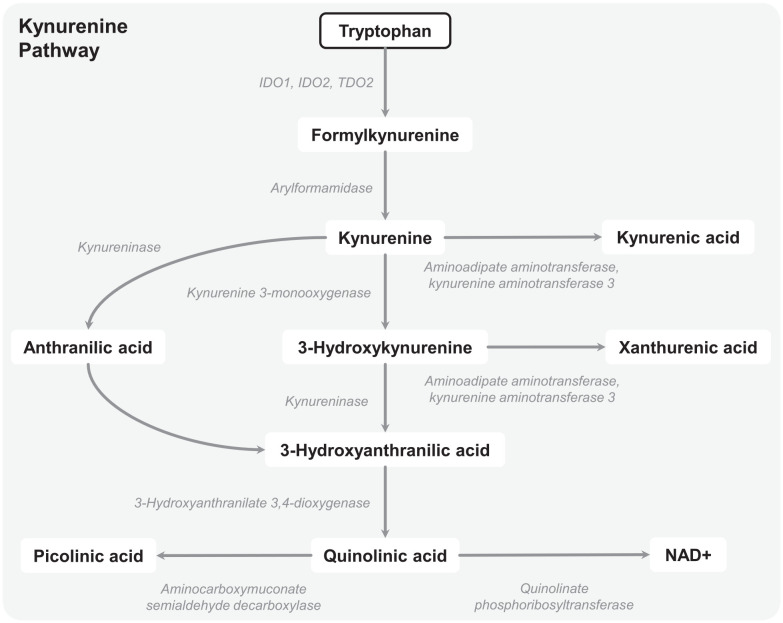
Overview of the kynurenine pathway (modified from: Broekhuizen et al^5^). Abbreviations: IDO, indoleamine 2,3-dioxygenase; TDO, tryptophan 2,3-dioxygenase; NAD^+^, nicotinamide adenine dinucleotide.

The KP is regulated by the hepatic tryptophan 2,3-dioxygenase (TDO)2, and the extrahepatic indoleamine 2,3-dioxygenase (IDO)1 and IDO2.^3,6^ These enzymes catalyze the conversion of L-tryptophan into N-formylkynurenine, which can be further metabolized into L-kynurenine, kynurenic acid, anthranilic acid, 3-hydroxy-anthranilic acid, quinolinic acid, picolinic acid, and nicotinamide adenine dinucleotide (NAD^+^) (Figure 1). In 1998, Munn et al^7^ revealed that inhibition of IDO resulted in pregnancy loss in mice, indicating that the KP is crucial to maintain pregnancy. The placenta is one of the few human tissues that constitutively expresses IDO1 under physiological conditions.^2,8^ Its expression and activity are reduced in pregnancies complicated by fetal growth restriction (FGR) and preeclampsia (PE).^5,9-12^

Under physiological conditions, total tryptophan concentrations decrease throughout pregnancy in maternal blood, while kynurenine concentrations remain constant.^13^ However, reference values of KP metabolites during pregnancy are currently lacking, and it is unclear how changes in tryptophan and kynurenine concentrations affect the downstream KP metabolites. Nevertheless, it is essential that KP metabolite concentrations are maintained within a certain range throughout pregnancy. This was demonstrated in animal studies in which tryptophan supplementation improved fetal growth and neonatal outcome, while excessive tryptophan intake led to a decreased placental and fetal weight and increased fetal mortality.^14-18^

Although the tryptophan metabolizing pathways toward melatonin and serotonin production have been implicated to play a role in pregnancy complications,^19-24^ little is yet known about how alterations in tryptophan metabolism into KP metabolites relate to pregnancy complications. Variations in KP metabolite concentrations as potential cause or consequence of pregnancy complications, may serve as novel biomarkers and/or (preventive) therapeutic targets. Therefore, this systematic review provides an overview of the current literature on KP metabolites variations during pregnancy in maternal blood, fetal blood, and the placenta in relation to maternal pregnancy and fetal outcomes.

## Methods

This systematic review was performed in accordance with the Preferred Reporting Items for Systematic Reviews and Meta-Analyses (PRISMA) guidelines,^25^ and the Meta-analysis of Observational Studies in Epidemiology (MOOSE) guidelines.^26^ The protocol was designed a priori and registered in PROSPERO, an international prospective register of systematic reviews (registration number: CRD42021273120).

### Search strategy, information sources, and eligibility criteria

A comprehensive literature search was performed in Embase, Medline, Web of Science, and Cochrane Central Register of Controlled Trials databases, including studies published before 18 February 2022. The full search strategy is shown in the Supplemental Appendix, but in short, it included synonyms of all KP metabolites, and terms related to the periconception and pregnancy periods, and maternal pregnancy and fetal outcomes.

Studies were eligible if KP metabolites were measured during the periconception period or pregnancy in maternal or fetal blood or in the placenta, and were related to maternal pregnancy or fetal outcomes. We included human studies written in the English language. Letters, editorials, opinion papers, case reports, case series, conference abstracts, and reviews were excluded.

### Study Selection and Data Extraction

Three independent reviewers (A.J.P.S. (initial search), M.B. (search update), and S.K.M.v.Z.) screened the title and abstract of unique records identified by the search. Next, the full texts of the selected studies were retrieved and assessed for final inclusion by two independent reviewers (M.B. and S.K.M.v.Z.). These 2 reviewers extracted the data from the included studies independently by using a pre-specified template. Throughout all stages of the selection and extraction processes, disagreements between the 2 reviewers were resolved by consensus or by consultation of a third reviewer (L.v.R.).

### Assessment of risk of bias

Two independent reviewers (M.B.and S.K.M.v.Z.) assessed the risk of bias using the ErasmusAGE quality score.^27,28^ This quality score consists of 5 items comprising study design (0 = cross-sectional, 1 = longitudinal, 2 = intervention), study size (0 = <100, 1 = 100-500, 2 = >500 participants), exposure (0  = not reported, 1 = moderate, 2 = adequate exposure measurement), outcome measurement (0 = not appropriate, 1 = moderate, 2 = adequate), and adjustments for confounders (0 = unadjusted, 1 = adjusted for key confounders, 2 = adjusted for additional covariates). This results in a quality score ranging between 0 and 10, with 10 representing the highest quality. The ErasmusAGE quality score is based on previously published scoring systems developed for *in vivo* clinical studies.^27,28^ However, no such scoring system exists for *ex vivo* studies.

### Data synthesis

We performed a narrative synthesis of the results of the included studies, grouped into maternal pregnancy and fetal outcomes. The direction of the associations between the KP metabolite concentrations and maternal pregnancy and fetal outcomes are presented in tables (Tables 2-6). The measures of effect were represented as in the original studies, and displayed as effect estimate (mean, median, *β*, or fold change (FC), with its respective error measure (standard deviation (SD), standard error (SE)), 95% confidence interval (95% CI), or interquartile range (IQR)), sample size (*N*) and *P*-value. If the measures of effect were not reported, the raw data (already available or provided upon request) were used to perform statistical analyses: linear regression analysis for continuous outcome variables, and an independent sample *t*-test to compare KP metabolite concentrations between 2 groups.

Since KP concentrations depend on the timing of sampling during pregnancy,^13^ and reference values during uncomplicated pregnancy are lacking, a meta-analysis was conducted of the means of KP metabolite concentrations per trimester of pregnancy with the condition that at least 3 studies reported absolute values of a specific KP metabolite in a similar matrix (maternal or fetal blood, or in the placenta). All statistical analyses were performed using SPSS (IBM SPSS Statistics 25) and R (R for Windows, version 3.5,^29^ R Package Meta^30^). A *P*-value < .05 was considered statistically significant.

## Results

### Study selection

**Figure 2.**
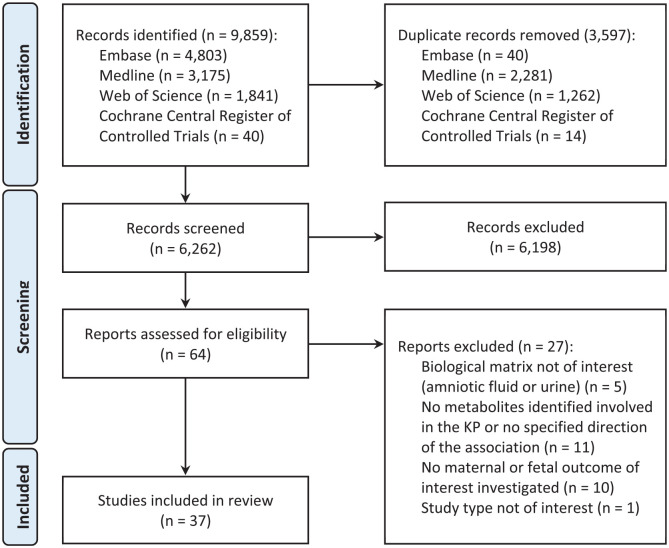
Flowchart of the process of literature search and selection of studies for this systematic review. Abbreviations: KP, kynurenine pathway.

The search identified 6262 unique records, of which 64 were found eligible for full-text reading after title and abstract screening. After reading the full texts, 37 studies were finally included (Figure 2).

### Study characteristics

The most important study characteristics are summarized in Table 1, showing that tryptophan and kynurenine were most frequently investigated compared to the other KP metabolites. A minority (n = 11) of the studies also measured other KP metabolites, including N-formylkynurenine, kynurenic acid, anthranilic acid, 3-hydroxykynurenine, xanthurenic acid, 3-hydroxyanthranilic acid, quinolinic acid, and picolinic acid. The KP metabolites were predominantly determined in maternal blood, but also in umbilical cord blood, and placental tissue. The KP metabolites were studied in relation to various maternal pregnancy and fetal outcomes. Maternal pregnancy outcomes included depression and anxiety during pregnancy, gestational diabetes mellitus (GDM), PE and pregnancy-induced hypertension (PIH), whereas fetal outcomes comprised FGR, birth weight, preterm birth (PTB), preterm premature rupture of membranes (PPROM), and spontaneous abortion (SA).

**Table 1.** Descriptive information of each included study.

Author	QS	Country	Sample size	Study design	Time period	Exposure	Outcome	Measured KP metabolites	Biological matrix	Timing of sampling
**Depression and anxiety during pregnancy**										
Sha et al^31^	7	USA	1st trimester = 122 2nd trimester = 88 3rd trimester = 82	Cohort	2015-2018	Depression	KP metabolite concentrations	Trp, Kyn, KA, QA, PA	Maternal plasma	1st, 2nd, and 3rd trimester
Keane et al^32^	6	Ireland	N = 209: IBS = 105 Control = 104	Cohort	Nov 2004-Jan 2011	Depression, anxiety	Trp and Kyn concentrations	Trp, Kyn	Maternal plasma	15 and 20 weeks
Groer et al^33^	5	USA	N = 374	Cross-sectional	NM	Depression	Trp and Kyn concentrations	Trp, Kyn	Maternal serum	2nd trimester
van Lee et al^34^	8	Singapore	N = 572	Cohort	Jun 2009-Sep 2010	Depression, anxiety	Trp and Kyn concentrations	Trp, Kyn	Maternal plasma	26-28 weeks
Nazzari et al^35^	6	Italy	N = 97	Cohort	NM	Depression, anxiety	KP metabolite concentrations	Trp, Kyn	Maternal serum	34-36 weeks
Teshigawara et al^36^	5	Japan	N = 132	Cohort	Oct 2012-Jan 2017	Depression	KP metabolite concentrations	Trp, Kyn, KA, AA, 3-HK, 3-HAA	Maternal plasma	3rd trimester
Scrandis et al^37^	3	USA	N = 27	Cohort	NM	Depression	Trp and Kyn concentrations	Trp, Kyn	Maternal serum	35-38 weeks
**Gestational diabetes mellitus**										
McMichael et al^38^	5	USA	N = 68: GDM = 34 non-GDM = 34	Case-control	NM	GDM	Metabolomic profile	Trp, Kyn, KA	Maternal plasma	10-16 weeks
Jiang et al^39^	8	China	N = 431	Cohort	Aug 2015-Jan 2016	GDM	Amino acid concentrations	Trp	Maternal serum	12-16 weeks
Zheng et al^40^	6	China	N = 60: GDM = 30 Control = 30	Case-control	NM	GDM	Metabolomic profile	Trp	Maternal plasma	20 weeks
Leitner et al^41^	4	Austria	N = 32: GDM = 14 Control = 18	Case-control	NM	GDM	Metabolomic profile	Trp	Maternal plasma, maternal urine	12-26 weeks
**Preeclampsia**										
Nilsen et al^42^	9	Norway	N = 2936	Cohort	Jul 2002-Dec 2003	PE	KP metabolite concentrations	Trp, Kyn, KA, AA, 3-HK, XA, 3-HAA	Maternal plasma	1st trimester
Jääskeläinen et al^43^	6	UK	N = 161: Early PE = 47 Control = 53 Late PE = 57 Control = 14	Cross-sectional case-control	2008-2011	PE	Metabolomic profile	Trp	Maternal serum	10-15 weeks, 23-41 weeks
Sander et al^44^	6	UK	N = 67: PE = 32 Control = 35	Case-control	NM	PE	Metabolomic profile	3-HAA	Maternal plasma	3rd trimester
Zhao et al^45^	4	China	N = 40: PE = 20 Control = 20	Case-control	NM	PE	KP metabolite concentrations	Trp, NFK, Kyn, KA, 3-HK, XA, 3-HAA, QA, PA, NAD+	Maternal serum, umbilical vein serum	Birth
Liu et al^46^	4	China	N = 38: PE = 14 Control = 24	Case-control	Jan 2015-Dec 2016	PE	Amino acid concentrations	Trp	Maternal blood, umbilical cord blood (dried blood spot)	Before delivery; birth
Kudo et al^10^	4	UK	N = 33: PE = 12 Pregnant = 12 Nonpregnant = 12	Case-control	NM	PE	Alterations in KP enzyme expression and activity, as well as KP metabolite concentrations	Trp, Kyn	Maternal plasma, placenta homogenates	3rd trimester
Broekhuizen et al^5^	NA	Netherlands	N = 57: PE = 18 Control = 39	*Ex vivo*	Jan 2018-Jan 2020	PE	Placental Trp metabolism, the effect of Trp on chorionic plate arteries	Trp, Kyn, KA, AA, 3-HK, XA, 3-HAA, QA	Placenta	Birth
Keaton et al^11^	NA	Sweden	N = 36: Late-onset PE = 18 Control = 18	*Ex vivo*	2003-2011	Late-onset PE	Trp, Kyn, and QA concentrations, the degree of expression and activity of the KP	Trp, Kyn, QA	Placenta	Birth
Zardoya-Laguardia et al^9^	NA	Austria	N = 82: FGR = 10 PE = 18 PTB = 10 Control = 44	*Ex vivo*	NM	PE, FGR	The effect of Trp on vasorelaxation chorionic plate arteries, and vessel back pressure of a placental cotyledon	Kyn	Placenta, chorionic plate arteries	Birth
Dunn et al^47^	NA	UK	N = 12: PE = 6 Control = 6	*Ex vivo*	NM	PE	Metabolomic profile	Kyn	Placental explant medium	Birth
**Pregnancy-induced hypertension**										
Ferranti et al^48^	7	USA	N = 100	Case-control	Jun 2014-Aug 2015	PE, PIH	Metabolomic profile	Kyn	Maternal serum	8-14 weeks
Grafka et al^49^	5	Poland	N = 210: PIH = 105 Control = 105	Case-control	2010-2014	PIH	Trp concentration	Trp	Maternal plasma	3rd trimester
Valensise et al^50^	4	Italy	N = 22: PIH = 20 Control = 12	Case-control	NM	PIH	Trp concentration	Trp	Maternal plasma, umbilical cord plasma	Birth
**Fetal growth (restriction)**										
Di Giulio et al^51^	5	Italy	N = 57: FGR = 8 1st trimester = 13 2nd trimester = 17 3rd trimester = 12 Control = 7	Case-control	NM	FGR, GA	Amino acid concentrations	Trp	Maternal plasma	1st, 2nd, and 3rd trimester
Robinson et al^52^	7	Belgium, Spain, Italy, Greece	N = 481	Cohort	(1) 2010-2013 (2) 2004-2006 (3) 2011-2013 (4) 2007-2008	Birthweight	Metabolomic profile	Methoxykynurenate (product of XA)	Umbilical cord plasma and serum	Birth
Moros et al^53^	5	Greece	N = 84: FGR = 48 Control = 36	Cross-sectional	NM	FGR	Metabolomic profile	Trp	Umbilical cord serum, maternal serum	Birth
Favretto et al^54^	5	Italy	N = 43: FGR = 22 AGA = 21	Cohort	Mar 2009-Dec 2009	FGR, GA	Metabolomic profile	Trp, Kyn	Umbilical vein serum	Birth
Cosmi et al^55^	4	Italy	N = 24: sFGR abnormal Dopplers = 4 sFGR normal Dopplers = 4 Control = 16	Case-control	Jan 2009-Jul 2011	sFGR	Metabolomic profile	Trp	Umbilical vein serum	Birth
Milart et al^56^	4	Poland	N = 32	Cross-sectional	NM	Birthweight, placental weight	KA concentration	KA	Maternal serum, umbilical cord serum	Birth
Horgan et al^57^	NA	UK	N = 17: SGA = 9 Control = 8	*Ex vivo*	NM	SGA	Metabolomic profile	Trp, Kyn	Placental explant medium	Birth
**Preterm birth**										
Li et al^58^	6	China	N = 101: RSA = 50 Control = 51	Case-control	Jan 2016-May 2017	RSA	Metabolomic profile	Kyn	Maternal serum	1st trimester
Guzel et al^59^	5	Turkey	N = 160	Cohort	Jan 2010-Aug 2010	PTB, birth weight	Amino acid concentrations	Trp	Maternal serum	1st trimester
Fei et al^60^	4	China	N = 30 (initial): MA = 15 Control = 15 N = 32 (validation): MA = 18 Control = 14	Case-control	Nov 2014-May 2015	MA	Metabolomic profile	Trp	Maternal serum	1st trimester
Virgiliou et al^61^	5	Greece	N = 70: PTB = 35 Control = 35	Case-control	NM	PTB	Metabolomic profile	Trp	Maternal serum, amniotic fluid	2nd trimester
Lizewska et al^62^	6	Poland	N = 143: PTB = 57 Threatened PTL = 49 Control = 25	Case-control	NM	PTB, threatened PTL, PPROM	Metabolomic profile	Trp	Maternal plasma	3rd trimester
Manuelpillai et al^63^	NA	Australia	N = 32: PPROM + infection = 8 PPROM - infection = 8 Control = 16	*Ex vivo*	NM	PPROM +/− infection	KP metabolite concentrations	Kyn, KA, 3-HAA, QA, PA	Umbilical vein blood, placental explant medium	3rd trimester

Abbreviations: Trp, tryptophan; Kyn, Kynurenine; KA, Kynurenic acid; NFK, N-formylkynurenine; AA, anthranilic acid; 3-HK, 3-hydroxykynurenine; XA, xanthurenic acid; 3-HAA, 3-hydroxyanthranilic acid; QA, quinolinic acid; PA, picolinic acid; AGA, appropriate for gestational age; EFW, estimated fetal weight; FGR, fetal growth restriction; GA, gestational age; GDM, gestational diabetes mellitus; IBS, inflammatory bowel syndrome; KP, kynurenine pathway; MA, missed abortion; NA, not applicable; NM, not mentioned; PE, preeclampsia; PIH, pregnancy-induced hypertension; PPROM, preterm premature rupture of membranes; PTB, preterm birth; PTL, preterm labor; QS, quality score; RSA, recurrent spontaneous abortion; sFGR, selective fetal growth restriction; SGA, small for gestational age.

Most of the studies were observational *in vivo* studies (n = 31), including case-control studies (n = 16), cohort studies (n = 11), and cross-sectional studies (n = 4). The 6 *ex vivo* studies investigated metabolism of tryptophan along the KP in placental tissue from PE or FGR pregnancies.^5,9,11,47,57,63^ In total 16 studies used metabolomics to identify underlying biological pathways and biomarkers in multiple pregnancy complications.^38,40,41,43,44,47,48,52-55,57,58,60-62^

**Figure 3.**
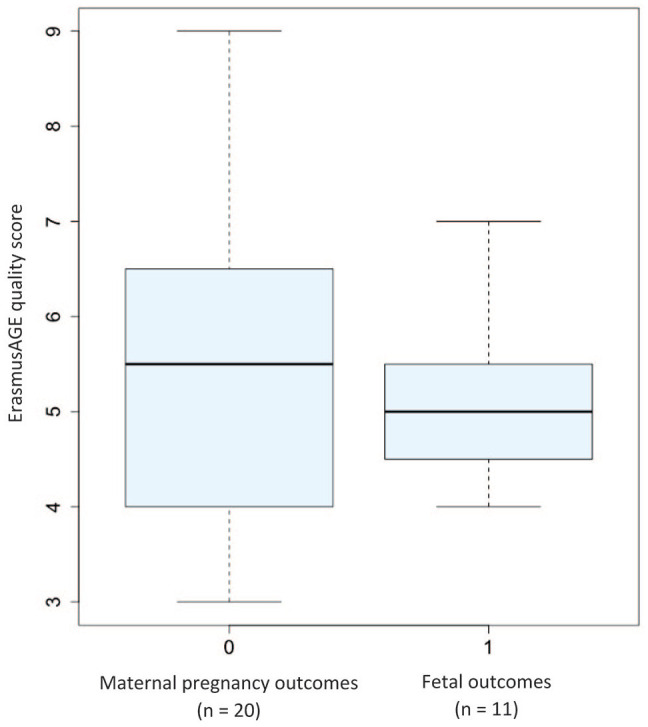
Boxplots of the ErasmusAGE quality score for the included *in vivo* studies grouped into studies investigating maternal pregnancy outcomes (depression and anxiety during pregnancy, gestational diabetes mellitus, preeclampsia, and pregnancy-induced hypertension; n = 20) and fetal outcomes (fetal growth restriction, birth weight, preterm birth, preterm premature rupture of membranes, spontaneous abortion; n = 11). The boxplots show the medians with interquartile ranges, the minimum and the maximum values.

The ErasmusAGE quality score of the *in vivo* studies ranged from 3 to 9, with a median of 5 (IQR = 4-6, Figure 3).

The boxplots show the medians with interquartile ranges, the minimum and the maximum values.

### Kynurenine Pathway Metabolite Concentrations in Uncomplicated Pregnancies

**Figure 4.**
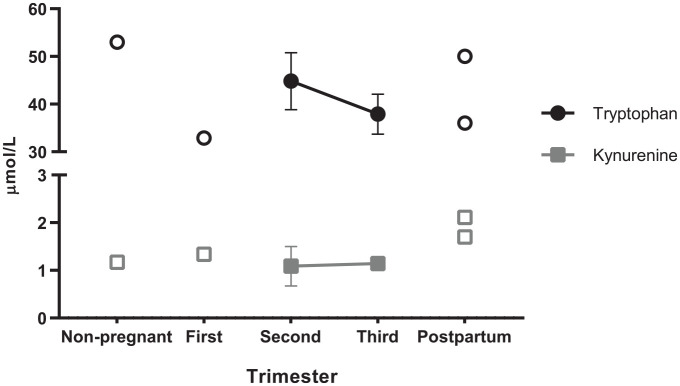
Tryptophan and kynurenine concentrations in healthy women, before pregnancy, as well as in the first trimester, second trimester, third trimester of pregnancy, and postpartum. The concentrations in the second and third trimester of pregnancy represent the pooled means ± standard error of tryptophan (N = 7) and kynurenine (N = 5), depicted as filled circles and squares, respectively. The open circles and squares represent values from single studies.

An overview of maternal tryptophan and kynurenine concentrations from the uncomplicated pregnancy populations in the included studies is given in Table 2. A meta-analysis could only be performed on the second and third trimester concentrations (Supplemental Figure 1). Figure 4 displays the pooled mean concentrations or, when not available, the concentrations from individual studies, including concentrations in non-pregnant state and postpartum. It can be concluded that the maternal tryptophan concentration decreases between the second and third trimester of pregnancy, while the maternal kynurenine concentration remains constant.

**Table 2.** Maternal tryptophan and kynurenine concentrations (µmol/L) throughout uncomplicated pregnancies per trimester of pregnancy.

Author	QS	N	Matrix	Fasting	Method of determination	Tryptophan, mean (SD)	Kynurenine, mean (SD)
*1st trimester*							
Sha et al^31^	7	90	Plasma	NM	HPLC + UV-detector	32.9 (5.4)	1.34 (0.33)
*2nd trimester*							
Nilsen et al^42^	9	2820	Plasma	No	GC-MS/MS, LC-MS/MS	59.0 (9.0)	1.11 (0.21)
Jiang et al^39^	8	366	Serum	Yes	UHPLC-MS/MS	43.4 (13.1)	
van Lee et al^34^	8	243	Plasma	Yes	LC-MS/MS	49.4 (8.2)	1.06 (0.20)
Sha et al^31^	7	76	Plasma	NM	HPLC + UV-detector	28.4 (4.3)	1.32 (0.17)
Keane et al^32^	6	104	Plasma	NM	HPLC + UV-/fluorescence-detector	32.5 (8.9)	0.99 (0.27)
Groer et al^33^	5	374	Serum	NM	HPLC + UV-/fluorescence-detector	62.6 (15.2)	1.90 (0.75)
Virgiliou et al^61^	5	35	Serum	NM	LC-MS	35.3 (6.2)	
*3rd trimester/at birth*							
Sha et al^31^	7	69	Plasma	NM	HPLC + UV-detector	28.4 (4.3)	1.32 (0.17)
Nazzari et al^35^	6	97	Serum	NM	HPLC + UV-/fluorescence-detector	54.4 (12.0)	1.00 (0.37)
Grafka et al^49^	5	105	Plasma	Yes	IEC + amino acid analyzer	35.0 (9.0)	
Zhao et al^45^	4	20	Serum	Yes	LC-MS/MS	34.5 (5.8)	0.85 (0.45)
Kudo et al^10^	4	12	Plasma	NM	HPLC + UV-detector	32.7 (4.8)	1.12 (0.17)
Valensise et al^50^	4	12	Plasma	NM	HPLC + UV-detector	35.6 (9.5)	
Scrandis et al^37^	3	27	Serum	NM	LC + UV-/fluorescence-detector	44.9 (9.5)	1.40 (0.40)

Abbreviations: GC, gas chromatography; HPLC, high-performance liquid chromatography; IEX, Ion-exchange chromatography; KP, kynurenine pathway; LC, liquid chromatography; MS, mass spectrometry; MS/MS, tandem mass spectrometry; NM, not mentioned; UHPLC, ultra-high-performance liquid chromatography; UV, ultraviolet.

### Maternal pregnancy outcomes

#### Depression and anxiety

##### Maternal blood

Seven studies (6 cohort, 1 cross-sectional) examined the association between KP metabolites and depressive symptoms (Table 3).^31-37^ Only one cohort study (QS = 7) determined KP metabolites in the first trimester of pregnancy, and found no associations with (the severity of) depressive symptoms.^31^

**Table 3.** Summary of studies that investigated associations between maternal KP metabolite concentrations and depression and anxiety during pregnancy.

Author	QS	Method	Association or comparison	Trp	Kyn	NFK	KA	AA	3-HK	XA	3-HAA	QA	PA
**Depression during pregnancy**													
*1st trimester*													
Sha et al^31^	7	Targeted	EPDS	=	=		=					=	=
*2nd trimester*													
Sha et al^31^	7	Targeted	EPDS	=	=		=					=	=
Keane et al^32^	6	Targeted	EPDS	=	=								
Groer et al^33^	5	Targeted	POMS-D > 20 vs POMS-D ⩽ 20	↓	=								
*3rd trimester*													
Sha et al^31^	7	Targeted	EPDS	=	=		=					↑	=
van Lee et al^34^	8	Targeted	EPDS	=									
Nazzari et al^35^	6	Targeted	EPDS	=	↓								
Teshigawara et al^36^	5	Targeted	Depression (EPDS) vs control	=	=		=	=	=		=		
Scrandis et al^37^	3	Targeted	SIGH-SAD	↓	=								
**Anxiety**													
*2nd trimester*													
van Lee et al^34^	8	Targeted	STAI	=									
Keane et al^32^	6	Targeted	STAI	=	=								
*3rd trimester*													
Nazzari et al^35^	6	Targeted	STAI	=	=								

Symbols: blank, not investigated or not identified in case of metabolomics; =, no association; ↓, negative association/lower concentration; ↑, positive association/higher concentration. Abbreviations: Trp, tryptophan; Kyn, Kynurenine; KA, Kynurenic acid; NFK, N-formylkynurenine; AA, anthranilic acid; 3-HK, 3-hydroxykynurenine; XA, xanthurenic acid; 3-HAA, 3-hydroxyanthranilic acid; QA, quinolinic acid; PA, picolinic acid; KP, kynurenine pathway; EPDS, Edinburgh Postnatal Depression Scale; POMS-D, Profile of Mood Status depression/dejection subscale; STAI, State-Trait Anxiety Inventory.

Of the 3 studies performed in the second trimester of pregnancy,^31-33^ one cross-sectional study (QS = 5) revealed lower tryptophan concentrations in women with a more depressed mood assessed by the depression/dejection subscale of the Profile of Mood Status (POMS-D; range 0-60), with a higher score indicating a more depressed mood (POMS-D scores >20 vs ⩽20 = 56.8 vs 63.2 µmol/L, N = 23 vs 351, *P* = .017).^33^ No such associations were found for kynurenine, kynurenic acid, quinolinic acid and picolonic acid.^31-33^

Out of 5 third trimester cohort studies,^31,34-37^ Scrandis et al^37^ (QS = 3) showed that tryptophan was negatively associated with depression (*β* = −.277, N = 27, *P* = .04), however, the other 4 larger studies did not confirm this.^31,34-36^ Interestingly, Scrandis et al^37^ assessed depressive symptoms using the structured Interview Guide for the Hamilton Depression Rating Scale-Seasonal Affective Disorder (SIGH-SAD), while the other studies used the more recently validated Edinburgh Postnatal Depression Scale (EPDS).^31,34-36^

Four of these third trimester cohort studies also investigated the association between kynurenine and depression.^31,35-37^ The results were conflicting, as these studies reported negative (QS = 6, *β* = −.002, SE = 0.001, *P* = .03)^35^, positive (QS = 7, EPDS ⩾ 13: OR (%) = 256.6, 95% CI = 21.3, 948.6, N = 82, *P* = .021),^31^ or no associations^34,36,37^ between kynurenine and depressive symptoms. Furthermore, Sha et al^31^ reported a positive association between quinolinic acid and (the severity of) depressive symptoms (QS = 7, total EPDS: OR (%) = 41.5, 95% CI = 1.8, 96.6, N = 82, *P* = .039; EPDS ⩾ 13: OR (%) 98.2, 95% CI = 10.4, 255.7, N = 82, *P* = .022). Kynurenic acid, anthranilic acid, 3-hydroxykynurenine, and 3-hydroxyanthranilic acid were not associated with (the severity of) depressive symptoms.^31,36^

Three cohort studies investigated tryptophan and kynurenine in relation to levels of anxiety.^32,34,35^ None of these studies found an association between tryptophan or kynurenine and anxiety symptoms during pregnancy. In all 3 studies the state of anxiety was measured using the State-Trait Anxiety Inventory (STAI).^64^

##### Summary

Low tryptophan concentrations in maternal blood in the second and third trimester of pregnancy may be associated with a more depressed mood during pregnancy. On the other hand, third-trimester quinolinic acid was positively associated with depression during pregnancy, while the other KP metabolites were not consistently altered in the second or third trimester of pregnancy. None of the studies observed an association between second- and third-trimester tryptophan and kynurenine and anxiety during pregnancy.

#### Gestational diabetes mellitus

Maternal blood. Four studies (1 cohort, 3 case-control) investigated KP metabolites in relation to GDM (Table 4).^38-41^ One case-control study (QS = 5) determined KP metabolites in the first trimester of pregnancy and suggested that kynurenine was elevated in women who developed GDM (FC = 1.42, GDM vs control N = 34 vs 34, *P* = .03).^38^ In these women, tryptophan, kynurenic acid and 3-hydroxyanthranilic acid concentrations were not altered.^38^

**Table 4.** Summary of studies that investigated associations between maternal KP metabolite concentrations and gestational diabetes mellitus.

Author	QS	Method	Comparison	Trp	Kyn	NFK	KA	AA	3-HK	XA	3-HAA	QA	PA
*1st trimester*													
McMichael et al^38^	5	Metabolomics	GDM vs control	=	↑		=				=		
*2nd trimester*													
Jiang et al^39^	8	Targeted	GDM	=									
Zheng et al^40^	6	Metabolomics	GDM vs control	↓									
Leitner et al^41^	4	Metabolomics	GDM vs control	↓									

Symbols: blank, not investigated or not identified in case of metabolomics; =, no association; ↓ negative association/lower concentration; ↑, positive association/higher concentration. Abbreviations: Trp, tryptophan; Kyn, Kynurenine; KA, Kynurenic acid; NFK, N-formylkynurenine; AA, anthranilic acid; 3-HK, 3-hydroxykynurenine; XA, xanthurenic acid; 3-HAA, 3-hydroxyanthranilic acid; QA, quinolinic acid; PA, picolinic acid; KP, kynurenine pathway; GDM, gestational diabetes mellitus.

The other 3 studies were performed in the second trimester of pregnancy.^39-41^ Two of these identified decreased tryptophan concentrations in women with GDM compared to controls through metabolomics (Zheng et al^40^: QS = 6, FC = 0.85, GDM vs. control N = 30 vs 30, *P* = .001; Leitner et al^41^: QS = 4, mean relative concentrations (SD) = 0.39 (0.28) vs 0.53 (0.35), GDM vs control N = 14 vs 18, *P* = .025 own analysis). However, Jiang et al^39^ (QS = 8) found no associations between tryptophan and GDM in a large cohort study. In all studies, GDM was diagnosed at 24 to 28 weeks of gestation using a routine oral glucose tolerance test (OGTT) and the International Association of Diabetes and Pregnancy Study Groups (IADPSG) criteria for diagnosis of GDM.^38-41^

#### Summary

A low maternal tryptophan concentration in the second trimester of pregnancy may be associated with GDM. This association was not found for other KP metabolites. Although data in the first trimester of pregnancy are limited, kynurenine might be positively associated with developing GDM.

#### Preeclampsia

Ten studies (5 case-control, 1 cohort, and 4 *ex vivo*) investigated associations between KP metabolites in maternal blood, fetal blood, and placental tissue and the development of PE (Table 5).^5,9-11,42-47^

**Table 5.** Summary of studies that investigated associations between maternal, fetal, and placental KP metabolite concentrations and hypertensive disorders of pregnancy.

Author	QS	Method	Association or comparison	Trp	Kyn	NFK	KA	AA	3-HK	XA	3-HAA	QA	PA
**Preeclampsia**													
*Maternal blood*													
*1st trimester*													
Nilsen et al^42^	9	Targeted	PE vs control	=	=		↑	=	=	=	=		
*2nd trimester*													
Jääskeläinen et al^43^	6	Metabolomics	PE vs control	=									
*3rd trimester*													
Sander et al^44^	6	Metabolomics	PE vs control								↑		
Zhao et al^45^	4	Targeted	PE vs control	=	=	=	=		=	=	=	=	=
			Proteinuria	=	=	=	+		=	=	=	=	+
Liu et al^46^	4	Targeted	PE vs control	=									
Kudo et al^10^	4	Targeted	Late-onset PE vs control	↑	=								
*Umbilical cord blood*													
Zhao et al^45^	4	Targeted	PE vs control	=	=	=	=		=	=	=	=	=
			Proteinuria	=	=	=	=		=	=	=	=	=
Liu et al^46^	4	Targeted	PE vs control	=									
*Placenta*													
Kudo et al^10^	4	Targeted	Late-onset PE vs control		↓								
Broekhuizen et al^5^	NA	Targeted	Early-onset PE vs control	↑	=		=	=	=	ND	=	=	
Keaton et al^11^	NA	Targeted	Late-onset PE vs control	↓	=							=	
Zardoya-Laguardia et al^9^	NA	Targeted	PE vs preterm control		↓								
Dunn et al^47^	NA	Metabolomics	PE vs control at 6% O_2_		↓								
**Pregnancy-induced hypertension**													
*Maternal blood*													
*1st trimester*													
Ferranti et al^48^	7	Metabolomics	PIH vs PE		↓								
*3rd trimester*													
Grafka et al^49^	5	Targeted	PIH vs control	↑									
Valensise et al^50^	4	Targeted	PIH vs control	=									
*Umbilical cord blood*													
Valensise et al^50^	4	Targeted	PIH vs control	=									

Symbols: blank, not investigated or not identified in case of metabolomics; =, no association; ↓, negative association/lower concentration; ↑, positive association/higher concentration; ND, not detectable. Abbreviations: Trp, tryptophan; Kyn, Kynurenine; KA, Kynurenic acid; NFK, N-formylkynurenine; AA, anthranilic acid; 3-HK, 3-hydroxykynurenine; XA, xanthurenic acid; 3-HAA, 3-hydroxyanthranilic acid; QA, quinolinic acid; PA, picolinic acid; KP, kynurenine pathway; PE, preeclampsia; PIH, pregnancy-induced hypertension.

##### Maternal blood

Only one study investigated the association between KP metabolites in the first trimester of pregnancy and PE, and found elevated kynurenic acid concentrations in women who later developed PE (QS = 9, mean (SE) = 0.0233 (0.00077) vs 0.0207 (0.00013) µmol/L, N = 2936, *P* < .001). At this stage of pregnancy, tryptophan and other KP metabolites were not altered.^42^

In women who had already developed PE in the third trimester of pregnancy, maternal kynurenic acid, as well as picolinic acid concentrations were positively associated with proteinuria (QS = 4, kynurenic acid: *r* = .684, N = 40, *P* < .025; picolinic acid: *r* = .641, N = 40, *P* < .031), suggesting a relation with severity of this disease. However, the rise in the concentrations of these metabolites was not large enough to result in statistically significant different concentrations between women with PE and uncomplicated pregnancies in this study.^45^ Most studies did also not identify altered tryptophan concentrations in women with PE in the third trimester of pregnancy (Zhao et al^45^: QS = 4, median (SE) = 37.0 (1.2) in PE vs 34.5 (1.3) in controls, N = 40, *P* ⩾ .05 ; Liu et al^46^: QS = 4, N = 38, *P* ⩾ .05; Jääskeläinen et al^43^: QS = 6, N = 71, *P* ⩾ .05). Only one study reported increased tryptophan in late-onset PE specifically (QS = 6, mean (SD): 42.8 (6.9) vs 32.7 (4.8) µmol/L, N = 33, *P* < .001).^10^ 3-Hydroxyanthranilic acid levels were elevated in women who had already developed PE in one metabolomics study (QS = 6, FC = 1.76, N = 67, *P* = .00014),^44^ but this was not confirmed by targeted analysis nor in another metabolomics study.^43,45^

##### Fetal blood

Concentrations of KP metabolites in the umbilical cord blood were similar between PE and uncomplicated pregnancies.^45,46^

##### Placenta

Placental concentrations of tryptophan were increased in early-onset PE (median (IQR) = 26.7 (20.6-30.2) vs 20.5 (15.7-24.1) ng/g tissue, N = 24, *P* = .005),^5^ and decreased in late-onset PE (mean (SD): 3.85 (0.88) vs 4.86 (1.30) µg/g tissue, N = 36, *P* = .01).^11^ Moreover, preeclamptic placentas secreted less kynurenine compared to healthy placentas *ex vivo*, measured by metabolomics (relative difference = 0.63, N = 12, *P* < .00005)^47^ as well as targeted analysis (Kudo et al^10^: 0.29 (0.04) vs 0.48 (0.06) nmol/mg/min, N = 22, *P* < .01 ; Zardoya-Laguardia et al^9^: N = 24, *P* ⩽ .05), implying reduced placental IDO1 activity.

##### Summary

Kynurenic acid was elevated in the first trimester of pregnancy in women with PE. Furthermore, both kynurenic acid and picolinic acid were positively associated with proteinuria in women with PE in the third trimester of pregnancy. None of the other KP metabolites was changed in maternal blood, nor was any KP metabolite altered in umbilical cord blood. Compared to healthy placentas, placental kynurenine production was lower in preeclamptic placentas, while the placental tryptophan concentration was increased in early-onset PE but decreased in late-onset PE.

#### Pregnancy-induced hypertension

##### Maternal blood

Two case-control studies investigated alterations in tryptophan concentrations in the third trimester in pregnancies complicated by PIH. In the largest of the 2 studies, tryptophan was significantly higher in women with PIH compared to controls (QS = 5, mean (SD): 99 (7) vs 35 (9) µmol/L, N = 210, *P* < .00005).^49^ However, in a smaller cohort study, this difference was not observed (QS = 4, mean (SD): 38.1 (10.3) vs 35.6 (9.5) µmol/L, N = 22).^50^

Although no studies were conducted to investigate variations of other KP metabolites in PIH specifically, the kynurenine concentration was lower in the first trimester in pregnant African American women who developed PIH compared to those who developed PE as identified through metabolomics (QS = 7, N = 100, *P* < .05).^48^

##### Fetal blood

No alterations of tryptophan were found in the umbilical cord blood of pregnancies complicated by PIH (mean (SD): 72.1 (16.8) vs 80.2 (19.6) µmol/L, N = 22).^50^

##### Summary

The tryptophan level is higher in women with PIH at the end of pregnancy compared to normotensive pregnant women.

### Fetal outcomes

#### Fetal growth restriction

Eight studies (3 cohort, 2 cross-sectional, 2 case-control, and 1 *ex vivo*) investigated the associations between KP metabolites in maternal blood, fetal blood, or placenta and FGR or birthweight (Table 6).^9,51-56,59^

**Table 6.** Summary of studies that investigated associations between maternal and fetal and placental KP metabolite concentrations and fetal outcomes.

QS	Author	QS	Method	Association or comparison	Trp	Kyn	NFK	KA	AA	3-HK	XA	3-HAA	QA	PA
**Fetal growth (restriction)**														
*Maternal blood*														
*1st trimester*														
Guzel et al^59^		5	Targeted	Birthweight	=									
Di Giulio et al^51^		5	Targeted	FGR vs control	=									
*3rd trimester*														
Moros et al^53^		5	Metabolomics	FGR vs control	↓									
Milart et al^56^		4	Targeted	Birthweight				=						
*Umbilical cord blood*														
Robinson et al^52^		7	Metabolomics	Birthweight	=						↓			
Moros et al^53^		5	Metabolomics	FGR vs control	↓									
Favretto et al^54^		5	Metabolomics	FGR vs control	↑	=								
Cosmi et al^55^		4	Metabolomics	sFGR twin vsAGA co-twin	↓									
Milart et al^56^		4	Targeted	Birthweight				=						
*Placenta*														
Zardoya-Laguardia et al^9^		NA	Targeted	FGR vs PTB		↓								
**Preterm birth**														
*Maternal blood*														
*1st trimester*														
Li et al^58^		6	Metabolomics	RSA vs control		↑								
Guzel et al^59^		5	Targeted	PTB vs control	=									
Fei et al^60^		4	Metabolomics	Missed abortion vs control	↓									
*2nd trimester*														
Virgiliou et al^61^		5	Metabolomics	PTB vs term	↓									
*3rd trimester*														
Lizewska et al^62^		6	Metabolomics	PTB vs term	=									
*Umbilical cord blood*														
Manuelpillai et al^63^		NA	Targeted	PPROM vs control		↓		↑						
*Placenta*														
Zardoya-Laguardia et al^9^		NA	Targeted	PTB vs term		↓								

Symbols: blank, not investigated or not identified in case of metabolomics; =, no association; ↓ negative association/lower concentration; ↑, positive association/higher concentration. Abbreviations: Trp, tryptophan; Kyn, Kynurenine; KA, Kynurenic acid; NFK, N-formylkynurenine; AA, anthranilic acid; 3-HK, 3-hydroxykynurenine; XA, xanthurenic acid; 3-HAA, 3-hydroxyanthranilic acid; QA, quinolinic acid; PA, picolinic acid; KP, kynurenine pathway; PTB, preterm birth; RSA, recurrent spontaneous abortion; MA, missed abortion; PPROM, preterm premature rupture of membranes.

##### Maternal blood

No statistically significant differences were observed in first-trimester tryptophan concentrations between women who did or did not carry a FGR child in two studies (QS = 5 for both).^51,59^ Although in adult pregnancies the first-trimester tryptophan concentration was not associated with low birthweight, it was associated with low birthweight in adolescent pregnancies (QS = 5, <2500 g, N = 39, *P* = .043).^59^ At birth tryptophan concentrations were also lower in women who carried a FGR child compared to uncomplicated pregnancies measured by metabolomics (QS = 5, mean (SD) µmol/L: 15.4 (11.4) vs 24.5 (7.1), N = 84, *P* < .001).^53^ However, the third-trimester kynurenic acid concentration was not related to birthweight in uncomplicated pregnancies (QS = 4).^56^

##### Fetal blood

Most data on umbilical cord blood variations in FGR were acquired using metabolomics and demonstrated conflicting results. One study reported a reduced tryptophan concentration in FGR fetuses (QS = 5, mean (SD) µmol/L: 18.1 (14.8) vs 35.6 (7.3), N = 84, *P* < .001 (own analysis of supplemental data)),^53^ and another study showed a trend toward a reduced tryptophan concentration in selective FGR twins compared to their appropriate-for-gestational-age co-twins (QS = 4, N = 20, no *P*-value reported).^55^ In contrast, a metabolomics study revealed higher tryptophan concentrations in FGR (QS = 5, N = 43, *P* < .0001) and found that tryptophan was an excellent discriminator between FGR and appropriate-for-gestational-age fetuses, while kynurenine was unaltered.^54^

Tryptophan was not associated with birthweight (QS = 7, N = 42),^52^ nor was kynurenic acid (QS = 4, N = 32).^56^ Only the isomeric form of methoxykynurenate, a product of xanthurenic acid, was negatively associated with birthweight (QS = 7, N = 42, *P* < .05).^52^

##### Placenta

Placental kynurenine formation, as measure for IDO1 activity, was significantly lower in FGR compared to preterm controls (N = 18, *P* ⩽ .01).^9^ A metabolomics study of the placental explant secretome revealed that with increasing O_2_ levels, the concentration of tryptophan decreased, while kynurenine increased in the medium of both explants from small for gestational age and appropriate-for-gestational-age fetuses.^57^

##### Summary

Although data on maternal KP metabolites in FGR were limited, low tryptophan concentrations in both maternal and fetal blood may be associated with FGR. Despite reduced placental kynurenine production in FGR, kynurenine seemed unaltered in fetal blood.

#### Preterm birth

##### Maternal blood

Two metabolomics studies reported a significant association between tryptophan metabolites in the first trimester of pregnancy and SA,^58,60^ a condition that may be considered an extreme form of PTB. While one of these studies found a decreased tryptophan concentration in SA (QS = 6, FC = 0.77, N = 32, *P* = .0026),^60^ kynurenine was found to be increased in the other study (QS = 4, FC = 1.41, N = 101, *P* = .04),^58^ but neither study confirmed each other’s finding.

Three studies (2 case-control, 1 cohort) investigated metabolomic profile and amino acid profile variations in relation to PTB.^59-61^ One metabolomics study found lower second-trimester tryptophan concentrations in women who gave birth prematurely (QS = 5, mean (SD) = 31.11 (5.52) vs 35.31 (6.19) µmol/L, N = 70, *P* = .0045).^61^ However, this association was not confirmed by the other 2 studies through self-reported dietary questionnaires in the first trimester of pregnancy (QS = 5, N = 160)^59^ or metabolomics in the third trimester of pregnancy before initiation of steroid or tocolytic therapy (QS = 6, N = 143).^62^ Also, third-trimester kynurenine concentrations were unaltered (QS = 6, N = 143).^62^

##### Fetal blood

Only one study investigated KP metabolites in umbilical cord blood in relation to PTB, in PPROM specifically. In PPROM with intrauterine infection kynurenine was decreased (*P* = .0019, N = 24), while kynurenic acid was increased (*P* = .0005, N = 24) when compared to term deliveries.^63^ Similar results were observed in PPROM without infection, although no statistics were mentioned. This study found no alterations in 3-hydroxyanthranilic acid, quinolinic acid, and picolinic acid concentrations.^63^

##### Placenta

Similar to the umbilical cord blood concentrations, *ex vivo* placental kynurenine formation was significantly lower in preterm compared to term controls (N = 20, *P* ⩽ .05).^9^

##### Summary

SA was associated with a lower tryptophan, but a higher kynurenine concentration in maternal blood in the first trimester of pregnancy compared to uncomplicated pregnancies. Similarly, the second-trimester tryptophan concentration was decreased in premature versus term pregnancies. The kynurenine concentration was lower in the premature-born placenta, and fetal blood of PPROM-pregnancies compared to controls.

## Discussion

**Figure 5.**
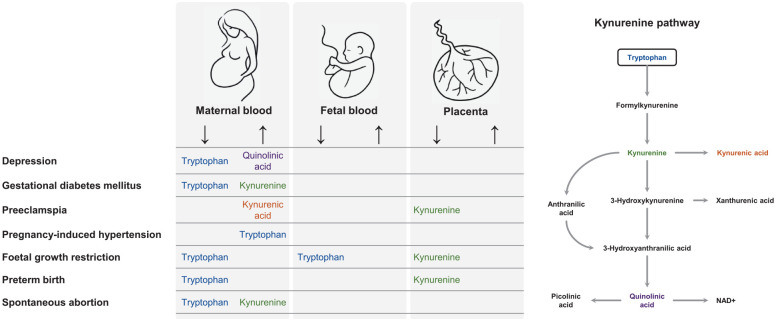
Summary of kynurenine pathway metabolite alterations in maternal and fetal blood, and in the placenta in relation to pregnancy complications. This figure focusses on kynurenine pathway metabolite alterations, and does not include unmeasured and undetected kynurenine pathway metabolites.

The present study summarized the associations between KP metabolite variations in maternal blood, fetal blood, and placental tissue, and maternal pregnancy and fetal outcomes (Figure 5). KP metabolites were mainly investigated in maternal blood, in the second and third trimester of pregnancy, while data on first-trimester KP metabolites were scarce. Compared to uncomplicated pregnancies, a low maternal tryptophan concentration was associated with depression, GDM, FGR, PTB, and SA, while a high tryptophan concentration was associated with PIH. Furthermore, a high kynurenic acid concentration in the first trimester of pregnancy was associated with developing PE. KP metabolites in fetal blood were investigated in relation to PE, PIH, FGR, and PTB, and only revealed a lower tryptophan concentration in FGR compared to appropriate-for-gestational-age fetuses. In the placenta, the kynurenine concentration and formation were attenuated in pregnancies complicated by PE, FGR, and PTB.

### Maternal pregnancy outcomes

#### Depression

In this study, we found that lower maternal tryptophan and higher maternal quinolinic acid concentrations in the second and third trimester of pregnancy may be related to severity of depression during pregnancy. Quinolinic acid is considered neurotoxic,^65,66^ and studies performed in non-pregnant populations also found associations between increased concentrations of quinolinic acid and depression.^67^ The decreased tryptophan and increased quinolinic acid concentrations in depression during pregnancy may, at least partly, be explained by changes in the gut microbiome, which was shown to regulate circulating KP metabolites, and was altered in patients with depressive disorders, but description of the underlying mechanisms falls beyond the scope of this review.^68-72^

#### Gestational diabetes mellitus

Two metabolomics studies reported decreased tryptophan concentrations in women with GDM,^40,41^ which was however not confirmed by the large cohort study of Jiang et al^39^ and the recent study of McMichael et al.^38^ The latter study did show an increased kynurenine concentration in women with GDM. Although results are ambiguous, potentially decreased tryptophan and increased kynurenine concentrations in maternal blood suggest an increased flux of tryptophan through the KP, possibly due to upregulation of IDO1 by the inflammatory state of GDM.^33,73^

#### Preeclampsia

An elevated kynurenic acid concentration in the first trimester of pregnancy before the onset of PE,^42^ and its correlation with proteinuria in women with PE^45^ could either be a consequence of early PE disturbances or an actual pathophysiological factor in PE. Although tryptophan and kynurenine concentrations were not altered in women with PE, the kynurenic acid concentration was elevated. Yet, kynurenine formation was attenuated in PE placentas. These discrepancies between placental and maternal KP changes indicate that the maternally elevated kynurenic acid concentration reflects KP alterations downstream of kynurenine and is unlikely a result of placental alterations.^2^ Instead, it might originate from another (yet unknown) source, and did not seem to affect the fetal kynurenic acid concentration.^45^

#### Pregnancy-induced hypertension

Given the lower kynurenine concentration in women with PIH versus PE as identified through metabolomics,^48^ and the similar concentration of kynurenine in PE and healthy women, it seems that women with PIH have both an increased tryptophan and a decreased kynurenine concentration.^49,50^ These data thus suggest a decreased flux of tryptophan through the KP in maternal blood in PIH which differs from PE, and potentially represents an altered activity of other KP degrading enzymes, such as hepatic TDO2.

### Fetal outcomes

#### Fetal growth restriction

Given that tryptophan is an essential amino acid and thus required for fetal growth, the relation between tryptophan supply and fetal growth is evident. Indeed, the tryptophan concentration was lower in the umbilical cord blood of fetuses with FGR compared to controls.^53,55^ Reduced maternal tryptophan concentrations in FGR pregnancies, though only observed at the end of pregnancy, corroborate with the hypothesis that insufficient maternal tryptophan intake can explain the lower fetal and maternal tryptophan concentrations in FGR pregnancies.^51,53,59^

#### Preterm birth

Women with SA and women with PTB both displayed lower tryptophan concentrations^58,60,61^ than women with term pregnancies. Low maternal tryptophan concentrations in PTB may affect fetal KP metabolites, but this remains subject for future studies. Maternal kynurenine concentrations were elevated in SA. It should be noted that in SA and PTB, KP metabolites have only been measured in maternal blood through metabolomics, or were calculated using self-reported dietary questionnaires, and therefore require more research.

### Placental kynurenine pathway metabolites

Placental conversion of tryptophan into kynurenine, representing IDO1 activity, was decreased in multiple human pregnancy complications including PE, FGR, PTB, and SA,^5,9,10,47,74,75^ suggesting that impaired KP flux may have a pathological role in human pregnancy complications.

Tryptophan can induce IDO1-dependent vasodilation in placental arteries, but in contrast to the decreased placental production of kynurenine by IDO1, vasodilation by tryptophan was enhanced in PE.^5^ A possible explanation for this observation might be that the placental KP function is determined by tryptophan transport rather than by IDO1 activity.^5,76^ Another potential explanation is that PE and FGR are both associated with placental insufficiency and hypothesized to encompass a hypoxic placental environment. A lower concentration of the IDO1 cofactor O_2_ was shown to reduce IDO1 expression^77^ and attenuate placental metabolism of tryptophan into kynurenine.^57^ Thus, this may compromise the formation of KP metabolites *in vivo*, in agreement with the reduced quinolinic acid formation in diet-induced FGR.^78^ As major source of *de novo* NAD^+^ formation, such a deficiency may contribute to insufficient placental development. Yet, this is contradicted by the observation that concentrations of the NAD^+^ precursor, quinolinic acid, were similar between PE and healthy placentas.^5,10^

Although in this review we specifically focused on tryptophan metabolism through the KP, it is important to acknowledge that KP alterations may also dysregulate the serotonin and melatonin pathways by changing tryptophan availability and aryl hydrocarbon receptor activation by kynurenine, and consequently affect mitochondrial function.^79,80^ Indeed, melatonin and serotonin were suggested to have a role in the pathogenesis of depression during pregnancy, GDM, PE, and FGR as well.^19-24^

### Strengths and limitations

This study is the first to provide a comprehensive overview of the current state of knowledge on variations of KP metabolites in complicated human pregnancies. Publication bias was limited by including all years of publication, performing quality assessment through the validated ErasmusAGE quality score,^27,28^ and by contacting corresponding authors directly for any unreported data and additional details relevant for the synthesis of the results. However, some publication bias might have arisen from the inclusion of metabolomics studies, since our search strategy did not find metabolomics studies that did not identify discriminatory alterations in KP metabolites. As a second limitation, heterogeneity in investigated KP metabolites maternal pregnancy and fetal outcomes complicated clustering of—and making equivalent comparisons between—results, limiting the possibilities of performing a meta-analysis. Thirdly, the included studies did not distinguish between free and total (albumin bound) tryptophan concentrations, while free tryptophan is available for transport to the fetus. Neither were free fatty acid concentrations measured, which are known to increase free tryptophan concentrations. Lastly, none of the included studies corrected for blood sampling seasonality, while the season can affect KP metabolite concentrations in pregnant women.^81^

## Conclusions and Implications

The KP might provide a diagnostically and therapeutically interesting target in complicated pregnancies, particularly in FGR where tryptophan seems to be decreased in both maternal and umbilical cord blood. Animal studies demonstrated that tryptophan supplementation improved embryo survival in mice exposed to pseudorabies virus-induced pregnancy failure,^14^ and fetal growth in ruminants,^15,16^ potentially through the role of KP metabolites in bone remodeling.^82^ Furthermore, the development of hypertension in the pups of rats with experimental chronic kidney disease was prevented by supplementing these pregnant rats with tryptophan.^17^

Before starting tryptophan supplementation, however, it is important to first investigate its effects on other KP metabolites. Our study showed that elevated kynurenic acid concentrations were associated with PE en PPROM, which could have detrimental neurodevelopment effects on the offspring.^83-89^ Thus, future studies should include longitudinal assessment of KP metabolites throughout (un)complicated pregnancies, and investigate the relation between KP metabolites in maternal and fetal blood.

Alterations in concentrations of KP metabolites do not necessarily correspond between maternal blood, fetal blood and placenta. Therefore, we believe it is time to revise the hypothesis that maternal KP metabolites reflect the placental KP and in particular placental IDO1 activity.

Kynurenic acid concentrations were elevated in maternal blood in PE and in the umbilical cord blood in PPROM, implying a potential pathological role for this KP metabolite. A decreased tryptophan concentration was observed in maternal blood in depression during pregnancy, GDM, FGR, PTB, and SA, and in fetal blood in FGR and PPROM, and was only found to be increased in PIH. Concurrently, the maternal concentration of kynurenine was lower in PIH and raised in GDM. Hence, while the flux of tryptophan through the KP seems enhanced in women with GDM, it may be attenuated in PIH. These data emphasize that physiological pregnancy requires a tight balance of KP metabolites, and that disturbances in either direction may be associated with adverse maternal pregnancy and fetal outcomes.

## Supplemental Material

sj-docx-1-try-10.1177_11786469221135545 – Supplemental material for The Role of the Kynurenine Pathway in the (Patho) physiology of Maternal Pregnancy and Fetal Outcomes: A Systematic Review Click here for additional data file. Supplemental material, sj-docx-1-try-10.1177_11786469221135545 for The Role of the Kynurenine Pathway in the (Patho) physiology of Maternal Pregnancy and Fetal Outcomes: A Systematic Review by Sofie KM van Zundert, Michelle Broekhuizen, Ashley JP Smit, Lenie van Rossem, Mina Mirzaian, Sten P Willemsen, AH Jan Danser, Yolanda B De Rijke, Irwin KM Reiss, Daphne Merkus and Régine PM Steegers-Theunissen in International Journal of Tryptophan Research

sj-docx-2-try-10.1177_11786469221135545 – Supplemental material for The Role of the Kynurenine Pathway in the (Patho) physiology of Maternal Pregnancy and Fetal Outcomes: A Systematic Review Click here for additional data file. Supplemental material, sj-docx-2-try-10.1177_11786469221135545 for The Role of the Kynurenine Pathway in the (Patho) physiology of Maternal Pregnancy and Fetal Outcomes: A Systematic Review by Sofie KM van Zundert, Michelle Broekhuizen, Ashley JP Smit, Lenie van Rossem, Mina Mirzaian, Sten P Willemsen, AH Jan Danser, Yolanda B De Rijke, Irwin KM Reiss, Daphne Merkus and Régine PM Steegers-Theunissen in International Journal of Tryptophan Research

sj-eps-3-try-10.1177_11786469221135545 – Supplemental material for The Role of the Kynurenine Pathway in the (Patho) physiology of Maternal Pregnancy and Fetal Outcomes: A Systematic Review Click here for additional data file. Supplemental material, sj-eps-3-try-10.1177_11786469221135545 for The Role of the Kynurenine Pathway in the (Patho) physiology of Maternal Pregnancy and Fetal Outcomes: A Systematic Review by Sofie KM van Zundert, Michelle Broekhuizen, Ashley JP Smit, Lenie van Rossem, Mina Mirzaian, Sten P Willemsen, AH Jan Danser, Yolanda B De Rijke, Irwin KM Reiss, Daphne Merkus and Régine PM Steegers-Theunissen in International Journal of Tryptophan Research

## References

[1] Bender DA. Biochemistry of tryptophan in health and disease. Mol Aspects Med. 1983;6:101-197. 6371429 10.1016/0098-2997(83)90005-5

[2] Broekhuizen M Danser AHJ Reiss IKM Merkus D. The function of the kynurenine pathway in the placenta: a novel pharmacotherapeutic target? Int J Environ Res Public Health. 2021;18:11545. 10.3390/ijerph182111545 PMC8582682 34770059

[3] Badawy AAB . Kynurenine pathway of tryptophan metabolism: regulatory and functional aspects. Int J Tryptophan Res. 2017;10:1178646917691938. 28469468 10.1177/1178646917691938 PMC5398323

[4] Badawy AA. The tryptophan utilization concept in pregnancy. Obstet Gynecol Sci. 2014;57:249-259. 25105097 10.5468/ogs.2014.57.4.249 PMC4124085

[5] Broekhuizen M Klein T Hitzerd E , et al. l-tryptophan-induced vasodilation is enhanced in preeclampsia: Studies on its uptake and metabolism in the human placenta. Hypertension. 2020;76:184-194. 32475317 10.1161/HYPERTENSIONAHA.120.14970

[6] Sedlmayr P Blaschitz A Stocker R. The role of placental tryptophan catabolism. Front Immunol. 2014;5:230. 24904580 10.3389/fimmu.2014.00230 PMC4032907

[7] Munn DH Zhou M Attwood JT , et al. Prevention of allogeneic fetal rejection by tryptophan catabolism. Science. 1998;281:1191-1193. 9712583 10.1126/science.281.5380.1191

[8] Yamazaki F Kuroiwa T Takikawa O Kido R. Human indolylamine 2,3-dioxygenase. Its tissue distribution, and characterization of the placental enzyme. Biochem J. 1985;230:635-638. 3877502 10.1042/bj2300635 PMC1152665

[9] Zardoya-Laguardia P Blaschitz A Hirschmugl B , et al. Endothelial indoleamine 2,3-dioxygenase-1 regulates the placental vascular tone and is deficient in intrauterine growth restriction and pre-eclampsia. Sci Rep. 2018;8:5488. 29615752 10.1038/s41598-018-23896-0 PMC5883010

[10] Kudo Y Boyd CA Sargent IL Redman CW. Decreased tryptophan catabolism by placental indoleamine 2,3-dioxygenase in preeclampsia. Am J Obstet Gynecol. 2003;188:719-726. 12634647 10.1067/mob.2003.156

[11] Keaton SA Heilman P Bryleva EY , et al. Altered tryptophan catabolism in Placentas from women with pre-eclampsia. Int J Tryptophan Res. 2019;12:1178646919840321. 31007529 10.1177/1178646919840321 PMC6457019

[12] Iwahashi N Yamamoto M Nanjo S Toujima S Minami S Ino K. Downregulation of indoleamine 2, 3-dioxygenase expression in the villous stromal endothelial cells of placentas with preeclampsia. J Reprod Immunol. 2017;119:54-60. 28131097 10.1016/j.jri.2017.01.003

[13] Schröcksnadel H Baier-Bitterlich G Dapunt O Wachter H Fuchs D. Decreased plasma tryptophan in pregnancy. Obstet Gynecol. 1996;88:47-50. 8684760 10.1016/0029-7844(96)00084-1

[14] Qiu S Fang Z Wu D Lin Y Che L. Tryptophan supplements promote pregnancy success in mice challenged with Pseudorabies Virus (PRV) by regulating the expression of systemic cytokines, immunoglobulins, PRV-specific protein profiles, and toll-like receptors. J Med Food. 2011;14:857-865. 21663516 10.1089/jmf.2010.1146

[15] Jo JH Lee JS Ghassemi Nejad J Kim WS Moon JO Lee HG. Effects of dietary supplementation of acetate and L-tryptophan conjugated bypass amino acid on productivity of pre- and post-partum dairy cows and their offspring. Animals. 2021;11:1726. 10.3390/ani11061726 PMC8226929 34207871

[16] Ma H Zhang W Zhu X Song W Liu J Jia Z. Effects of rumen-protected tryptophan on growth performance, fibre characteristics, nutrient utilization and plasma essential amino acids in cashmere goats during the cashmere slow growth period. Livestock Sci. 2010;131:227-233.

[17] Hsu CN Lin IC Yu HR Huang LT Tiao MM Tain YL. Maternal tryptophan supplementation protects adult rat offspring against hypertension programmed by maternal chronic kidney disease: implication of tryptophan-metabolizing microbiome and aryl hydrocarbon receptor. Int J Mol Sci. 2020;21:4552. 32604820 10.3390/ijms21124552 PMC7349830

[18] Tsuji A Nakata C Sano M Fukuwatari T Shibata K. L-tryptophan metabolism in pregnant mice fed a high L-tryptophan diet and the effect on maternal, placental, and fetal growth. Int J Tryptophan Res. 2013;6:21-33. 24009424 10.4137/IJTR.S12715 PMC3748091

[19] Lanoix D Guérin P Vaillancourt C. Placental melatonin production and melatonin receptor expression are altered in preeclampsia: new insights into the role of this hormone in pregnancy. J Pineal Res. 2012;53:417-425. 22686298 10.1111/j.1600-079X.2012.01012.x

[20] Berbets AM Barbe AM Andriiets OA Andriiets AV Yuzko OM. Melatonin levels decrease in the umbilical cord in case of intrauterine growth restriction. J Med Life. 2020;13:548-553. 33456605 10.25122/jml-2020-0128 PMC7803309

[21] de Melo IMF Ferreira CGM Alves ÉR , et al. Melatonin administration prevents placental and fetal changes induced by gestational diabetes. Reprod Sci. 2022;29:1111-1123. 35025098 10.1007/s43032-022-00850-0

[22] Martínez-Paredes JF Jácome-Pérez N. Depression in pregnancy depresión en el embarazo. Rev Colomb Psiquiatr (Engl Ed). 2019;48:58-65. 30651174 10.1016/j.rcp.2017.07.003

[23] Bolte AC van Geijn HP Dekker GA. Pharmacological treatment of severe hypertension in pregnancy and the role of serotonin(2)-receptor blockers. Eur J Obstet Gynecol Reprod Biol. 2001;95:22-36. 11267716 10.1016/s0301-2115(00)00368-7

[24] Ranzil S Ellery S Walker DW , et al. Disrupted placental serotonin synthetic pathway and increased placental serotonin: potential implications in the pathogenesis of human fetal growth restriction. Placenta. 2019;84:74-83. 31176514 10.1016/j.placenta.2019.05.012 PMC6724713

[25] Page MJ McKenzie JE Bossuyt PM , et al. The PRISMA 2020 statement: an updated guideline for reporting systematic reviews. BMJ. 2021;372:n71. 10.1136/bmj.n71 PMC8005924 33782057

[26] Stroup DF Berlin JA Morton SC , et al. Meta-analysis of observational studies in epidemiology: a proposal for reporting. Meta-analysis of observational studies in Epidemiology (MOOSE) group. JAMA. 2000;283:2008-2012. 10789670 10.1001/jama.283.15.2008

[27] Carter P Gray LJ Troughton J Khunti K Davies MJ. Fruit and vegetable intake and incidence of type 2 diabetes mellitus: systematic review and meta-analysis. BMJ. 2010;341:c4229-c4229. 10.1136/bmj.c4229 PMC2924474 20724400

[28] Thomas BH Ciliska D Dobbins M Micucci S. A process for systematically reviewing the literature: providing the research evidence for public health nursing interventions. Worldviews Evid Based Nurs. 2004;1:176-184. 17163895 10.1111/j.1524-475X.2004.04006.x

[29] Core Team. A Language and Environment for Statistical Computing. R Foundation for Statistical Computing; 2022. http://www.R-project.org/

[30] Balduzzi S Rücker G Schwarzer G. How to perform a meta-analysis with R: a practical tutorial. Evid Based Ment Health. 2019;22:153-160. 31563865 10.1136/ebmental-2019-300117 PMC10231495

[31] Sha Q Madaj Z Keaton S , et al. Cytokines and tryptophan metabolites can predict depressive symptoms in pregnancy. Transl Psychiatry. 2022;12:35. 35078975 10.1038/s41398-022-01801-8 PMC8789799

[32] Keane JM Khashan AS McCarthy FP , et al. Identifying a biological signature of prenatal maternal stress. JCI Insight. 2021;6:143007. 33301421 10.1172/jci.insight.143007 PMC7934857

[33] Groer M Fuchs D Duffy A Louis-Jacques A D’Agata A Postolache TT. Associations among obesity, inflammation, and tryptophan catabolism in pregnancy. Biol Res Nurs. 2018;20:284-291. 29141444 10.1177/1099800417738363 PMC6346309

[34] van Lee L Cai S Loy SL , et al. Relation of plasma tryptophan concentrations during pregnancy to maternal sleep and mental well-being: the GUSTO cohort. J Affect Disord. 2018;225:523-529. 28866296 10.1016/j.jad.2017.08.069 PMC5667743

[35] Nazzari S Molteni M Valtorta F Comai S Frigerio A. Prenatal IL-6 levels and activation of the tryptophan to kynurenine pathway are associated with depressive but not anxiety symptoms across the perinatal and the post-partum period in a low-risk sample. Brain Behav Immun. 2020;89:175-183. 32531426 10.1016/j.bbi.2020.06.015

[36] Teshigawara T Mouri A Kubo H , et al. Changes in tryptophan metabolism during pregnancy and postpartum periods: potential involvement in postpartum depressive symptoms. J Affect Disord. 2019;255:168-176. 31158779 10.1016/j.jad.2019.05.028

[37] Scrandis DA Langenberg P Tonelli LH , et al. Prepartum depressive symptoms correlate positively with C-reactive protein levels and negatively with tryptophan levels: a preliminary report. Int J Child Health Hum Dev. 2008;1:167-174. 18924606 PMC2567806

[38] McMichael LE Heath H Johnson CM , et al. Metabolites involved in purine degradation, insulin resistance, and fatty acid oxidation are associated with prediction of gestational diabetes in plasma. Metabolomics. 2021;17:105. 34837546 10.1007/s11306-021-01857-5 PMC8741304

[39] Jiang R Wu S Fang C , et al. Amino acids levels in early pregnancy predict subsequent gestational diabetes. J Diabetes. 2020;12:503-511. 31883199 10.1111/1753-0407.13018

[40] Zheng S Zhong J Chen Y , et al. Metabolic profiling of plasma in gestational diabetes mellitus using liquid chromatography and Q-TOF Mass spectrometry. Clin Lab. 2017;63:1045-1055. 28792694 10.7754/Clin.Lab.2017.161110

[41] Leitner M Fragner L Danner S , et al. Combined metabolomic analysis of plasma and urine reveals AHBA, tryptophan and serotonin metabolism as potential risk factors in gestational diabetes mellitus (GDM). Front Mol Biosci. 2017;4:84. 29312952 10.3389/fmolb.2017.00084 PMC5742855

[42] Nilsen RM Bjørke-Monsen AL Midttun O , et al. Maternal tryptophan and kynurenine pathway metabolites and risk of preeclampsia. Obstet Gynecol. 2012;119:1243-1250. 22617590 10.1097/AOG.0b013e318255004e PMC3360419

[43] Jääskeläinen T Kärkkäinen O Jokkala J , et al. A non-targeted LC-MS metabolic profiling of pregnancy: longitudinal evidence from healthy and pre-eclamptic pregnancies. Metabolomics. 2021;17:20. 33515103 10.1007/s11306-020-01752-5 PMC7846510

[44] Sander KN Kim DH Ortori CA , et al. Untargeted analysis of plasma samples from pre-eclamptic women reveals polar and apolar changes in the metabolome. Metabolomics. 2019;15:157. 31773355 10.1007/s11306-019-1600-8 PMC6879453

[45] Zhao YJ Zhou C Wei YY , et al. Differential distribution of tryptophan-metabolites in fetal and maternal circulations during normotensive and preeclamptic pregnancies. Reprod Sci. 2022;29:1278-1286. 34622427 10.1007/s43032-021-00759-0 PMC8917071

[46] Liu G Deng W Cui W , et al. Analysis of amino acid and acyl carnitine profiles in maternal and fetal serum from preeclampsia patients. J Matern -Fetal Neonatal Med. 2020;33:2743-2750. 30563378 10.1080/14767058.2018.1560407

[47] Dunn WB Brown M Worton SA , et al. Changes in the metabolic footprint of placental explant-conditioned culture medium identifies metabolic disturbances related to hypoxia and Pre-Eclampsia. Placenta. 2009;30:974-980. 19775752 10.1016/j.placenta.2009.08.008

[48] Ferranti EP Frediani JK Mitchell R , et al. Early pregnancy serum metabolite profiles associated with hypertensive disorders of pregnancy in African American Women: A Pilot Study. J Pregnancy. 2020;2020:1515321. 32148965 10.1155/2020/1515321 PMC7049834

[49] Grafka A Łopucki M Karwasik-Kajszczarek K Stasiak-Kosarzycka M Dzida G. Plasma concentration of tryptophan and pregnancy-induced hypertension. Arterial Hypertens. 2018;22:9-15.

[50] Valensise H Scartozzi A Gianantoni D , et al. Materno-fetal concentration of tryptophan and fetal behavioural states in normal and PIH pregnancies. Preliminary results. Clin Exp Hypertens Part B Hypertens Pregnancy. 1988;7:253-259.

[51] Di Giulio AM Carelli S Castoldi RE Gorio A Taricco E Cetin I. Plasma amino acid concentrations throughout normal pregnancy and early stages of intrauterine growth restricted pregnancy. J Matern Fetal Neonatal Med. 2004;15:356-362. 15280104 10.1080/14767050410001725578

[52] Robinson O Keski-Rahkonen P Chatzi L , et al. Cord blood metabolic signatures of birth weight: A population-based study. J Proteome Res. 2018;17:1235-1247. 29401400 10.1021/acs.jproteome.7b00846

[53] Moros G Boutsikou T Fotakis C , et al. Insights into intrauterine growth restriction based on maternal and umbilical cord blood metabolomics. Sci Rep. 2021;11:7824. 33837233 10.1038/s41598-021-87323-7 PMC8035183

[54] Favretto D Cosmi E Ragazzi E , et al. Cord blood metabolomic profiling in intrauterine growth restriction. Anal Bioanal Chem. 2012;402:1109-1121. 22101423 10.1007/s00216-011-5540-z

[55] Cosmi E Visentin S Favretto D , et al. Selective intrauterine growth restriction in monochorionic twin pregnancies: Markers of endothelial damage and metabolomic profile. Twin Res Hum Genet. 2013;16:816-826. 23701694 10.1017/thg.2013.33

[56] Milart P Urbanska EM Turski WA Paszkowski T Sikorski R. Intrapartum levels of endogenous glutamate antagonist-kynurenic acid in amniotic fluid, umbilical and maternal blood. Neurosci Res Commun. 1999;24:173-178.

[57] Horgan RP Broadhurst DI Dunn WB , et al. Changes in the metabolic footprint of placental explant-conditioned medium cultured in different oxygen tensions from placentas of small for gestational age and normal pregnancies. Placenta. 2010;31:893-901. 20708797 10.1016/j.placenta.2010.07.002

[58] Li X Yin M Gu J Hou Y Tian F Sun F. Metabolomic profiling of plasma samples from women with recurrent spontaneous abortion. Med Sci Monit. 2018;24:4038-4045. 29898462 10.12659/MSM.907653 PMC6031124

[59] Guzel AI Cinar M Erkilinc S , et al. Association between adverse perinatal outcomes and amino acid levels measured with nutrient questionnaire in adolescent pregnancies. J Chin Med Assoc. 2016;79:335-339. 27071985 10.1016/j.jcma.2015.12.008

[60] Fei H Hou J Wu Z , et al. Plasma metabolomic profile and potential biomarkers for missed abortion. Biomed Chromatogr. 2016;30:1942-1952. 27229294 10.1002/bmc.3770

[61] Virgiliou C Gika HG Witting M , et al. Amniotic fluid and maternal serum metabolic signatures in the second trimester associated with preterm delivery. J Proteome Res. 2017;16:898-910. 28067049 10.1021/acs.jproteome.6b00845

[62] Lizewska B Teul J Kuc P , et al. Maternal plasma metabolomic profiles in spontaneous preterm birth: Preliminary results. Mediators Inflamm. 2018;2018:9362820. 29670470 10.1155/2018/9362820 PMC5833472

[63] Manuelpillai U Ligam P Smythe G Wallace EM Hirst J Walker DW. Identification of kynurenine pathway enzyme mRNAs and metabolites in human placenta: up-regulation by inflammatory stimuli and with clinical infection. Am J Obstet Gynecol. 2005;192:280-288. 15672037 10.1016/j.ajog.2004.06.090

[64] Marteau TM Bekker H. The development of a six-item short-form of the state scale of the Spielberger State-Trait Anxiety Inventory (STAI). Br J Clin Psychol. 1992;31:301-306. 1393159 10.1111/j.2044-8260.1992.tb00997.x

[65] Stone TW Perkins MN. Quinolinic acid: a potent endogenous excitant at amino acid receptors in CNS. Eur J Pharmacol. 1981;72:411-412. 6268428 10.1016/0014-2999(81)90587-2

[66] Guillemin GJ. Quinolinic acid, the inescapable neurotoxin. FEBS J. 2012;279:1356-1365. 22248144 10.1111/j.1742-4658.2012.08485.x

[67] Maes M Leonard BE Myint AM Kubera M Verkerk R. The new '5-HT' hypothesis of depression: cell-mediated immune activation induces indoleamine 2,3-dioxygenase, which leads to lower plasma tryptophan and an increased synthesis of detrimental tryptophan catabolites (TRYCATs), both of which contribute to the onset of depression. Prog Neuropsychopharmacol Biol Psychiatry. 2011;35:702-721. 21185346 10.1016/j.pnpbp.2010.12.017

[68] O’Mahony SM Clarke G Borre YE Dinan TG Cryan JF. Serotonin, tryptophan metabolism and the brain-gut-microbiome axis. Behav Brain Res. 2015;277:32-48. 25078296 10.1016/j.bbr.2014.07.027

[69] Sampson TR Mazmanian SK. Control of brain development, function, and behavior by the microbiome. Cell Host Microbe. 2015;17:565-576. 25974299 10.1016/j.chom.2015.04.011 PMC4442490

[70] Jiang H Ling Z Zhang Y , et al. Altered fecal microbiota composition in patients with major depressive disorder. Brain Behav Immun. 2015;48:186-194. 25882912 10.1016/j.bbi.2015.03.016

[71] Kaur H Bose C Mande SS. Tryptophan metabolism by gut microbiome and gut-brain-axis: an in silico analysis. Front Neurosci. 2019;13:1365. 10.3389/fnins.2019.01365 PMC6930238 31920519

[72] Lukić I Getselter D Koren O Elliott E. Role of tryptophan in microbiota-induced depressive-like behavior: evidence from tryptophan Depletion Study. Front Behav Neurosci. 2019;13:123. 31231198 10.3389/fnbeh.2019.00123 PMC6558209

[73] Pantham P Aye IL Powell TL. Inflammation in maternal obesity and gestational diabetes mellitus. Placenta. 2015;36:709-715. 25972077 10.1016/j.placenta.2015.04.006 PMC4466145

[74] Karahoda R Robles M Marushka J , et al. Prenatal inflammation as a link between placental expression signature of tryptophan metabolism and preterm birth. Hum Mol Genet. 2021;30:2053-2067. 34169316 10.1093/hmg/ddab169 PMC8561419

[75] Ban Y Chang Y Dong B Kong B Qu X. Indoleamine 2,3-dioxygenase levels at the normal and recurrent spontaneous abortion fetal-maternal interface. J Int Med Res. 2013;41:1135-1149. 23847296 10.1177/0300060513487642

[76] Kudo Y Boyd CA. The role of L-tryptophan transport in L-tryptophan degradation by indoleamine 2,3-dioxygenase in human placental explants. J Physiol. 2001;531:417-423. 11230514 10.1111/j.1469-7793.2001.0417i.x PMC2278460

[77] Murthi P Wallace EM Walker DW. Altered placental tryptophan metabolic pathway in human fetal growth restriction. Placenta. 2017;52:62-70. 28454699 10.1016/j.placenta.2017.02.013

[78] Sano M Ferchaud-Roucher V Kaeffer B Poupeau G Castellano B Darmaun D. Maternal and fetal tryptophan metabolism in gestating rats: Effects of intrauterine growth restriction. Amino Acids. 2016;48:281-290. 26334345 10.1007/s00726-015-2072-4

[79] Mazzoccoli G Kvetnoy I Mironova E , et al. The melatonergic pathway and its interactions in modulating respiratory system disorders. Biomed Pharmacother. 2021;137:111397. 33761613 10.1016/j.biopha.2021.111397

[80] Anderson G Maes M. Gut dysbiosis dysregulates central and systemic homeostasis via subop-timal mitochondrial function: assessment, treatment and classification implications. Curr Top Med Chem. 2020;20:524-539. 32003689 10.2174/1568026620666200131094445

[81] Levitan RD Sqapi M Atkinson L , et al. Seasonality of plasma tryptophan and kynurenine in pregnant mothers with a history of seasonal affective disorder: vulnerability or adaptation? World J Biol Psychiatr. 2020;21:529-538. 10.1080/15622975.2020.1769189 32462949

[82] Michalowska M Znorko B Kaminski T Oksztulska-Kolanek E Pawlak D. New insights into tryptophan and its metabolites in the regulation of bone metabolism. J Physiol Pharmacol. 2015;66:779-791. 26769827

[83] Buck SA Baratta AM Pocivavsek A. Exposure to elevated embryonic kynurenine in rats: sex-dependent learning and memory impairments in adult offspring. Neurobiol Learn Mem. 2020;174:107282. 32738461 10.1016/j.nlm.2020.107282 PMC7506508

[84] Rentschler KM Baratta AM Ditty AL , et al. Prenatal kynurenine elevation elicits Sex-Dependent changes in sleep and arousal during adulthood: Implications for psychotic disorders. Schizophr Bull. 2021;47:1320-1330. 33823027 10.1093/schbul/sbab029 PMC8379538

[85] Beggiato S Notarangelo FM Sathyasaikumar KV Giorgini F Schwarcz R. Maternal genotype determines kynurenic acid levels in the fetal brain: Implications for the pathophysiology of schizophrenia. J Psychopharmacol. 2018;32:1223-1232. 30354938 10.1177/0269881118805492

[86] Baratta AM Kanyuch NR Cole CA Valafar H Deslauriers J Pocivavsek A. Acute sleep deprivation during pregnancy in rats: rapid elevation of placental and fetal inflammation and kynurenic acid. Neurobiol Stress. 2020;12:100204. 32258253 10.1016/j.ynstr.2019.100204 PMC7109515

[87] Pershing ML Phenis D Valentini V , et al. Prenatal kynurenine exposure in rats: age-dependent changes in NMDA receptor expression and conditioned fear responding. Psychopharmacology. 2016;233:3725-3735. 27527585 10.1007/s00213-016-4404-9 PMC5808405

[88] Forrest CM McNair K Pisar M Khalil OS Darlington LG Stone TW. Altered hippocampal plasticity by prenatal kynurenine administration, kynurenine-3-monoxygenase (KMO) deletion or galantamine. Neuroscience. 2015;310:91-105. 26365611 10.1016/j.neuroscience.2015.09.022 PMC4642643

[89] Hahn B Reneski CH Pocivavsek A Schwarcz R. Prenatal kynurenine treatment in rats causes schizophrenia-like broad monitoring deficits in adulthood. Psychopharmacology. 2018;235:651-661. 29128872 10.1007/s00213-017-4780-9 PMC5823752

