# Supplementary material for: The Role of the Kynurenine Pathway in the (Patho) physiology of Maternal Pregnancy and Fetal Outcomes: A Systematic Review
Source: Int J Tryptophan Res. 2022 Nov 30;15:11786469221135545. doi: 10.1177/11786469221135545 (PMC9716456; doi:10.1177/11786469221135545)
Supplement: sj-docx-2-try-10.1177_11786469221135545 – Supplemental material for The Role of the Kynurenine Pathway in the (Patho) physiology of Maternal Pregnancy and Fetal Outcomes: A Systematic Review [file sj-docx-2-try-10.1177_11786469221135545.docx]

**Supplementary Material**

**Search**

*Embase*

(Trp/de OR 'Trp metabolism'/de OR 'Trp deficiency'/de OR Kyn/de OR 'kynurenic acid'/de OR 'xanthurenic acid'/de OR 'quinolinic acid'/de OR 'quinolinic acid derivative'/de OR 'picolinic acid'/de OR 'picolinic acid derivative'/de OR '3 hydroxyanthranilic acid'/de OR 'nicotinamide adenine dinucleotide'/de OR (Trp* OR LevoTrp* OR Kynurenin* OR Formylkynurenin* OR Kynurenic-Acid* OR Kynurenate* OR HydroxyKyn* OR Xanthurenic-Acid* OR Anthranilic-Acid* OR Anthranilat* OR Anthranilat* OR o-Aminobenzoic-Acid* OR 2-Aminobenzoic-Acid* OR Vitamin-L1 OR Vitamin-L-1 OR 2-Aminobenzoate* OR o-Aminobenzoate* OR Quinolinic-Acid* OR Quinolinate* OR Pyridinecarboxylic-Acid* OR Pyridine-2-Carboxylic-Acid* OR Hydroxyanthranilic-Acid* OR Hydroxyanthranilate* OR OH-Anthranilic-Acid*):Ab,ti) AND (pregnancy/exp OR 'pregnancy disorder'/exp OR 'prenatal development'/exp OR 'birth weight'/exp OR 'parameters concerning the fetus, newborn and pregnancy'/exp OR 'embryonic and placental structures'/exp OR 'perinatal period'/de OR embryo/exp OR fetus/exp OR 'prenatal period'/exp OR 'prenatal exposure'/exp OR ((crown NEAR/3 rump) OR pregnant* OR pregnanc* OR Gravid* OR gestation* OR prenatal* OR perinatal* OR embryo* OR fetus* OR foetus* OR fetal* OR foetal* OR placenta* OR Eclampsia* OR Preeclampsia* OR (Edema NEAR/3 Proteinuria NEAR/3 Hypertension NEAR/3 Gestosis) OR HELLP* OR Intrauterin* OR Intra-uterin* OR Morning-Sickness OR Preterm* OR Pre-term* OR prematurity OR ((prematur* OR term) NEAR/3 (born OR birth OR labor OR labour)) OR birthweight* OR birth-weight* OR Preconception* OR Periconception* OR Antenatal* OR Trimester*):Ab,ti) NOT [conference abstract]/lim AND [english]/lim

*Medline*

(Trp/ OR Kyn/ OR Kynurenic Acid/ OR Xanthurenates/ OR Quinolinic Acid/ OR Picolinic Acids/ OR 3-Hydroxyanthranilic Acid/ OR NAD/ OR (Trp* OR LevoTrp* OR Kynurenin* OR Formylkynurenin* OR Kynurenic-Acid* OR Kynurenate* OR HydroxyKyn* OR Xanthurenic-Acid* OR Anthranilic-Acid* OR Anthranilat* OR Anthranilat* OR o-Aminobenzoic-Acid* OR 2-Aminobenzoic-Acid* OR Vitamin-L1 OR Vitamin-L-1 OR 2-Aminobenzoate* OR o-Aminobenzoate* OR Quinolinic-Acid* OR Quinolinate* OR Pyridinecarboxylic-Acid* OR Pyridine-2-Carboxylic-Acid* OR Hydroxyanthranilic-Acid* OR Hydroxyanthranilate* OR OH-Anthranilic-Acid*).ab,ti.) AND (exp Pregnancy/ OR exp Pregnancy Complications/ OR exp Birth Weight/ OR Crown-Rump Length/ OR Diabetes, Gestational/ OR Embryonic Development/ OR Fetal Development/ OR Fetal Growth Retardation/ OR Hyperemesis Gravidarum/ OR Hypertension, Pregnancy-Induced/ OR Infant, Small for Gestational Age/ OR exp Embryonic Structures/ OR Perinatology/ OR Maternal Exposure/ OR exp Prenatal Exposure Delayed Effects/ OR ((crown ADJ3 rump) OR pregnant* OR pregnanc* OR Gravid* OR gestation* OR prenatal* OR perinatal* OR embryo* OR fetus* OR foetus* OR fetal* OR foetal* OR placenta* OR Eclampsia* OR Preeclampsia* OR (Edema ADJ3 Proteinuria ADJ3 Hypertension ADJ3 Gestosis) OR HELLP* OR Intrauterin* OR Intra-uterin* OR Morning-Sickness OR Preterm* OR Pre-term* OR prematurity OR ((prematur* OR term) ADJ3 (born OR birth OR labor OR labour)) OR birthweight* OR birth-weight* OR Preconception* OR Periconception* OR Antenatal* OR Trimester*).ab,ti.) AND english.la.

*Web of Science*

TS=(((Trp* OR LevoTrp* OR Kynurenin* OR Formylkynurenin* OR Kynurenic-Acid* OR Kynurenate* OR HydroxyKyn* OR Xanthurenic-Acid* OR Anthranilic-Acid* OR Anthranilat* OR Anthranilat* OR o-Aminobenzoic-Acid* OR 2-Aminobenzoic-Acid* OR Vitamin-L1 OR Vitamin-L-1 OR 2-Aminobenzoate* OR o-Aminobenzoate* OR Quinolinic-Acid* OR Quinolinate* OR Pyridinecarboxylic-Acid* OR Pyridine-2-Carboxylic-Acid* OR Hydroxyanthranilic-Acid* OR Hydroxyanthranilate* OR OH-Anthranilic-Acid*)) AND (((crown NEAR/2 rump) OR pregnant* OR pregnanc* OR Gravid* OR gestation* OR prenatal* OR perinatal* OR embryo* OR fetus* OR foetus* OR fetal* OR foetal* OR placenta* OR Eclampsia* OR Preeclampsia* OR (Edema NEAR/2 Proteinuria NEAR/2 Hypertension NEAR/2 Gestosis) OR HELLP* OR Intrauterin* OR Intra-uterin* OR Morning-Sickness OR Preterm* OR Pre-term* OR prematurity OR ((prematur* OR term) NEAR/2 (born OR birth OR labor OR labour)) OR birthweight* OR birth-weight* OR Preconception* OR Periconception* OR Antenatal* OR Trimester*))) AND DT=(article) AND LA=(english)

Cochrane Central Register of Controlled Trials

((Trp* OR LevoTrp* OR Kynurenin* OR Formylkynurenin* OR Kynurenic NEXT Acid* OR Kynurenate* OR HydroxyKyn* OR Xanthurenic NEXT Acid* OR Anthranilic NEXT Acid* OR Anthranilat* OR Anthranilat* OR o NEXT Aminobenzoic NEXT Acid* OR 2 NEXT Aminobenzoic NEXT Acid* OR Vitamin NEXT L1 OR Vitamin NEXT L NEXT 1 OR 2 NEXT Aminobenzoate* OR o NEXT Aminobenzoate* OR Quinolinic NEXT Acid* OR Quinolinate* OR Pyridinecarboxylic NEXT Acid* OR Pyridine NEXT 2 NEXT Carboxylic NEXT Acid* OR Hydroxyanthranilic NEXT Acid* OR Hydroxyanthranilate* OR OH NEXT Anthranilic NEXT Acid*):Ab,ti) AND (((crown NEAR/3 rump) OR pregnant* OR pregnanc* OR Gravid* OR gestation* OR prenatal* OR perinatal* OR embryo* OR fetus* OR foetus* OR fetal* OR foetal* OR placenta* OR Eclampsia* OR Preeclampsia* OR (Edema NEAR/3 Proteinuria NEAR/3 Hypertension NEAR/3 Gestosis) OR HELLP* OR Intrauterin* OR Intra NEXT uterin* OR Morning NEXT Sickness OR Preterm* OR Pre NEXT term* OR prematurity OR ((prematur* OR term) NEAR/3 (born OR birth OR labor OR labour)) OR birthweight* OR birth NEXT weight* OR Preconception* OR Periconception* OR Antenatal* OR Trimester*):Ab,ti)
